# Effect of an Emergency Department Closure on Homeless Patients and Adjacent Hospitals

**DOI:** 10.5811/westjem.2021.12.53918

**Published:** 2022-04-05

**Authors:** Scott Gummerson, Megan Smith, Otis Warren

**Affiliations:** * Johns Hopkins Hospital, Department of Emergency Medicine, Baltimore, Maryland; † Boston University School of Social Work, Boston, Massachusetts; ‡ The Miriam Hospital, Department of Emergency Medicine, Providence, Rhode Island

## Abstract

**Introduction:**

Homeless and housed patients differ on several emergency department (ED) metrics (emergency medical services [EMS] use, chief complaints, admission rates, etc.). On January 1, 2018, Memorial Hospital (MH), a safety-net hospital in Pawtucket, Rhode Island, closed. We studied the impact of this closure by analyzing homeless patient utilization of the two closest EDs before and after MH closed.

**Methods:**

A retrospective chart review compared the ED records of The Miriam Hospital (TMH), (1.8 miles from MH) and Rhode Island Hospital (RIH), (4.3 miles from MH). We analyzed visits between January 1, 2017–December 30, 2018. (MH closed on 1/1/2018). Patients were identified as homeless if their address listed was either “homeless” or a shelter/homeless service provider. All other patients were assumed to be housed. We removed from the analysis visits without an address listed or visits missing other key study variables (1.6% of the total).

**Results:**

A total of 113,925 unique patients visited the RIH and TMH EDs in 2017, as well as 117,167 in 2018. Homeless patients accounted for 1.18% of patients seen in 2017 and 1.32% in 2018. Between 2017 and 2018, this represents an increase of individual homeless patients of 15.46% (1553-1345), while the number of unique housed patients increased by 2.69% (115,614-112,580). The closer hospital, TMH, saw a 43.72% increase in homeless visits, while RIH saw an 8% increase. Homeless patients were discharged significantly more often than housed patients (74% vs 65%) and had significantly longer time to admission (466.0 vs 304.0 minutes) and discharge (397.9 vs 263.7 minutes) compared to housed patients. Homeless patients presented with suicidality (8.61% of visits) and alcohol-related concerns (29.88% of visits) significantly more than housed patients (1.43% and 2.94%, respectively).

**Conclusion:**

When a local ED closes, other EDs are impacted. We found visits made by homeless patients increased more than those made by housed patients and skewed significantly toward the closer hospital. We also found that homeless patients spend significantly more time in the ED and presented with behavioral health complaints more frequently. This impact of hospital closure on patterns of ED utilization by homeless patients has implications for ED management and homeless services both in the ED and the community.

## INTRODUCTION

Annual emergency department (ED) utilization has increased dramatically in the past two decades, growing from approximately 107.5 million visits in 2001 to over 145.5 million in 2016.^1^ As hospitals strain to keep up with increases in demand, EDs across the country continue to experience greater wait times, lengths of stay, and difficulty adjusting to increased utilization.^2^–^5^ Compounding these issues, EDs are being closed faster than they are being opened, resulting in a decreasing number of EDs tasked with an increasingly larger population of patients.^6^,^7^ Additionally, hospitals that care for uninsured and impoverished patients are more likely to close than others, leading to disproportionately adverse health outcomes for vulnerable populations.^6^–^9^

Homeless individuals are one such population that disproportionately shoulders the detrimental effects of local ED closure. Hospitals that care for uninsured and vulnerable people are at a greater risk of closing, and homeless individuals are known to access emergency services at higher rates (per capita) than non-homeless individuals.^7^,^10^,^11^ As local hospitals close, homeless individuals may also face greater logistical issues when attempting to access care at EDs that remain open, which may be geographically farther away and require transportation to access.^8^,^9^,^12^ This introduces additional challenges for a population that already faces substantial barriers to care, and may ultimately lead to poor health outcomes.^9^,^13^–^15^ Given that this population historically has higher psychiatric and substance use-related health needs, increasing these services at remaining hospitals would be an important adaptation to a changing patient population. Nevertheless, the effect of local ED closure on homeless individuals has not been explicitly or adequately explored in recent literature. Here we study the implications of ED closure for homeless populations and their effect on remaining hospitals.

## METHODS

We conducted a retrospective chart review to examine the impact of a naturally occurring experiment to examine the differences in the frequency and characteristics of ED visits by homeless patients at two hospitals before and after a third local hospital closed.

### Study Settings

Memorial Hospital, which ceased operations on January 1, 2018, was a community hospital located in the city of Pawtucket, RI. It had a single, family medicine residency program. While the ED and inpatient units closed, the outpatient family medicine clinic remained open. From 2011–2017, MH was responsible for 37% of all ED visits in its service area, as well as 6.22% of all ED visits in the state.^16^ A report published by the RI Department of Health showed that the MH ED cared for a large portion of the state’s vulnerable population, and that its closure may have detrimentally affected the local population and remaining operational EDs.^16^,^17^ Memorial Hospital did not have any specific housing resources, and there were no significant changes in the available housing or social services in the area after its closure.

This report found that MH’s patient population was more chronically ill, more impoverished, less educated, more likely to be a racial minority, more likely to use ED services, and more likely to be uninsured than patients in the rest of the state.^16^ Moreover, they were less likely to own a car or have access to transportation.^16^ The catchment area of MH was predominantly from the cities of Pawtucket and Central Falls, RI. These cities are lower income areas with higher poverty rates compared with the rest of the state. According to 2020 census data, Pawtucket had a median household income of $50,476, and 15.6% of the population lived below the poverty level. Central Falls had a mean household income of $32,982, and 30.2% of the population lived below the poverty level.

**Population Health Research Capsule**

What do we already know about this issue?

*Memorial Hospital (MH) cared for many vulnerable patients before closing. These patients were more likely to be poor or uninsured compared to the rest of Rhode Island.*

What was the research question?

*How did MH’s closure affect emergency department (ED) utilization by homeless individuals at nearby EDs that remained open?*

What was the major finding of the study?

*Nearby EDs saw a disproportionate increase in visits by homeless individuals compared to the non-homeless.*

How does this improve population health?

*When safety-net hospitals close, nearby EDs absorb their patients and should consider increasing social services to account for increases in homeless visits.*

We analyzed data of the two closest hospitals before and after MH’s closure; the Miriam Hospital (TMH) (1.8 miles from MH) and Rhode Island Hospital (4.3 miles from MH). Both these hospitals are in the neighboring city of Providence, RI. The Miriam Hospital, located in the East Side neighborhood of Providence, is a 247-bed academic/community hospital affiliated with a medical school and trains medical students, residents, and fellows in multiple specialties. It does not have a specialized area in the ED for care of psychiatric or intoxicated patients, nor does it have an inpatient psychiatric or detoxification unit. The East Side neighborhood has a significantly higher median household income level of $100,631. Rhode Island Hospital (4.3 miles from MH) is a 719-bed, Level I trauma center that trains medical students, residents, and fellows in multiple specialties; RIH has a specialized psychiatric and intoxication unit within the ED and an inpatient psychiatric unit. It is located in South Providence, which has a lower median household income level of $34,053.

Rhode Island does not have a geographically centralized location of homeless or social services, although these tend to be concentrated in the metro center. Because all three hospitals in this study (MH, TMH, and RIH) are also in the greater Providence area, these resources are located throughout their catchment areas.

While homeless patients certainly made up a subset of MH’s vulnerable patient population, homeless individuals were not explicitly studied in the RI Department of Health or recent research on the subject.^16^,^17^ The purpose of this paper was to use this naturally occurring experiment to investigate how the closure of MH may have affected ED utilization by homeless individuals in state. Here, we attempt to describe the changes in ED utilization by homeless individuals at two large, nearby EDs in Rhode Island, TMH and RIH. in the years before and after MH closed.

### Chart Abstraction

This chart review was conducted by a hospital-affiliated data abstractor who was blinded to the study’s objectives and hypothesis, and who used a self-created Epic chart review algorithm (Epic Systems Inc., Verona, WI) to extract data from all ED visits at TMH and RIH between January 1, 2017–December 30, 2018 (MH closed on 1/1/2018). The information extracted included a “homeless flag” if the patient’s address was listed either as “homeless” or as one of the recognized homeless shelters or service providers in the areas. Both hospitals use the same method for address recording. This information was provided to the extractor by one of the authors (MS) who has knowledge of homeless services in the area. Other extracted information included the hour of patient arrival, the arrival hospital’s name, the arrival method, chief complaint for the visit, ED disposition, number of visits, gender, age, ethnicity, race, insurance/payer financial class, length of stay (from arrival to departure), and door-to-disposition time (which is the time recorded from when the patient presented to ED to the time a disposition was entered).

We defined inclusion criteria as any visit to the RIH or TMH EDs from 2017 to 2018, while exclusion criteria were defined as any visit that had the housing field left blank or was otherwise missing key study variables. Included in the final analysis were 9414 homeless patient visits and 343,912 housed patient visits for a total of 353,326 visits (98.40%) (Figure). We removed 3476 (<1%) visits from the analysis due to missing address field, and we also omitted 2,280 (<1%) visits missing key study variables (Figure). Repeat visits for both housed and homeless patients were counted toward the total number of visits for each year.

The abstracted data represents a total population of patients at the two hospitals; thus, no statistical analysis of this data was performed, as summary means and proportions were calculated directly. The Lifespan Institional Review Board approved this study.

## RESULTS

Between both hospitals (TMH and RIH) there were 359,083 total visits during the two-year study period, of which 353,326 included sufficient data to be included in the analysis (Table 1). Of these visits, 343,912 were made by housed patients and 9,414 were made by homeless patients. The demographics of the housed and homeless patients are listed in Table 1. Homeless patients arrived by emergency medical services (EMS) more frequently (52% vs 30%), and had longer ED stays both when they were admitted (466.0 vs 304.0 minutes) or discharged (397.9 vs 263.7 minutes) compared to housed patients (Table 2). Homeless patients also presented with suicidality (8.61% vs 1.43%) and for alcohol-related visits (29.66% vs 2.94%) at increased rates when compared to housed patients.

We found the percentage of homeless patients seen at TMH and RIH increased in the year after the closure of MH, from 1.18% of all patients in 2017 to 1.32% in 2018 (Table 3). This represents an 11.86% increase in homeless patients seen. At TMH, the closer hospital (1.8 miles away), total homeless patient increased a total of 43.72% (279 to 401 homeless patients), while the number of unique housed patients increased by 8.82% (47,055 to 51,203). At RIH, the homeless patients increased 8.07% over this period of time, while the number of unique housed patients decreased by −1.70%. During the study period, unique housed patients made an average of 1.5 visits per year at both hospitals combined, while homeless patients made an average of 2.25 visits.

## DISCUSSION

Our study demonstrates homeless patients were disproportionately affected by the closing of a local hospital. As the homeless population in Rhode Island did not significantly change between 2018 to 2019 and there were no significant changes in housing or primary care resources, our findings of disproportionate increases of homeless visits at the remaining hospitals were directly related to MH’s closure rather than to any other factors.^18^

This study evaluates 1) how the presence of a local ED affects the surrounding homeless population, and 2) how a local homeless population affects the operations of an ED. We used a hospital’s closure to examine the effect on homeless patients and their impact on the closest remaining hospitals. A naturally occurring experiment in Rhode Island happened when MH closed on January 1, 2018. Our data shows that while the volume at the two closest remaining EDs increased modestly, the number of homeless patients increased drastically, particularly at the closer facility. The extent and the nuances of this increased volume is critically dependent on specific local factors, including proximity of other hospitals, patient makeup, and resources.

We found that homeless patients were disproportionately affected compared to housed patients. Specifically, the number of homeless patients seen at the remaining hospitals increased 11.86%%, while the number of housed patients increased only 2.69%. Our data speaks to the important role a local ED has in the life of a homeless person. We found the admission rates to be significantly less for homeless patients, suggesting that an ED acts as a critical access point for homeless patients.

Our data also suggests that when a local hospital closed, the number of homeless patients seen increased proportionately more at the next closest hospital (TMH), even if it is only marginally closer than others and does not have specialized care areas for psychiatric and substance use-related disorders. However, homeless patients seen increased by a greater absolute number at the larger, Level I trauma center that offered more specialized care in substance use and psychiatry. We found that compared to housed patients, homeless patients are more reliant on EMS. Perhaps going to the closest hospital is not a choice that the homeless person is making but is a decision made for them by the EMS personnel in the ambulance that takes them, or by what facilities are in walking distance or accessible via city bus lines. Moreover, our data suggests that a local ED functions as a necessary resource for homeless patients, and decisions about where to receive healthcare is made for them by local infrastructure.

We found homeless patients have longer ED lengths of stay, a higher rate of repeat visits, and higher rates of suicidality and alcohol-related visits, which is consistent with prior work.^19^–^22^ Therefore, when a hospital sees an increase in the proportion of homeless patients, we can expect many metrics of ED processes to be affected (patient flow, and psychiatric and substance use disorder resources). This is exactly what happened. Lawrence et al described overall increases in ED utilization, wait times, lengths of stay, and patients who left without being seen at TMH and RIH, in the year after MH’s closure.^17^ While these changes may be due to overall increases in non-homeless patients (2.69%), it more likely represents an increased burden from a disproportionately greater number of homeless patients (a 11.86% increase).

The two remaining hospitals (TMH and RIH), while both teaching hospitals of the same medical school and run by the same parent organization, saw distinct changes after MH closed. The Miriam Hospital, the closer hospital to the closed MH, is in an affluent neighborhood. Our data indicates that MH acted as a buffer of sorts for TMH. When MH closed, TMH saw a 43.72% increase in the number of homeless patients in the year after this buffer was lifted. The RIH also saw an increase in the number of homeless patients. While the percentage increase in homeless patients was less than TMH, RIH saw a greater absolute number of homeless patients after MH closed.

These two remaining hospitals were different to begin with. The RIH, which is Rhode Island’s only Level I trauma center, is located in a neighborhood with high poverty rates and saw a large number of homeless patients even before MH closed. By contrast, TMH saw comparatively fewer homeless patients. Our findings suggest that when a hospital closes, a smaller hospital that sees fewer homeless patients should expect the greatest percentage of change, particularly if that hospital is closer to the closing hospital. However, hospitals that already treat higher numbers of homeless patients should expect these numbers to increase. Additionally, smaller hospitals without inpatient psychiatry or substance use services should anticipate the greater need for these services, more than larger hospitals where these services already exist.

As hospital closures across the country are increasing in frequency, our data can serve as a case example for the remaining local hospitals, demonstrating that they should expect to see increased patient volume and ought to adjust for the likely disproportionately increased numbers of homeless patients.^6^,^7^ We found that overall this could mean implementing increased social services at the remaining hospitals, including housing first and substance use services, which have been shown to decrease ED and EMS utilization.^23^–^26^ Furthermore, coordination of services provided at the city and state level should consider these findings, as these hospital closures will likely have a downstream effect on local healthcare and social service utilizations in general.

## LIMITATIONS

Limitations of our study include the local nature of our assessment, and the fact that the unique circumstances of any state’s hospital system, political makeup, or particular homeless population make it difficult to generalize. Additionally, as the influence of EMS diversion on ED volumes was not explored, it is unclear how EMS protocols may have influenced the distribution of homeless patients to local hospitals—especially as this population was shown to use EMS services at greater rates.

We used a given address as a proxy for housing status in our retrospective work. This likely led to an undercounting of homeless patients, as they would not have been captured using this methodology if they gave a former address or that of a friend or family member. Additionally, we found a number of patients had blank address fields. While the overall percentage of these visits was small, we could not verify the housing status of visits based upon the retrospective design of our study. Furthermore, homeless patients who listed the address of family or a friend would be considered as not homeless in our study, further undercounting our homeless patients.

We reviewed records from the two closest remaining hospitals (TMH and RIH) that shared an electronic health record. We did not review records from other hospitals that could have also been affected because there was no access to this data and these hospitals were further away from the closed hospital (MH). Additionally, we only followed trends one year after the hospital closure. There may be trends that are longer or more sustained that this study did not evaluate. Future research should study the effects of local ED closures on homeless populations in other areas of the country, and in different hospital systems to determine whether our findings are replicated elsewhere. Additional investigation is also needed to see whether increased services for homeless individuals at remaining operational EDs (and from city and state governments) could preemptively alleviate the effect of local ED closure on homeless individuals and surrounding hospitals.

## CONCLUSION

When a hospital and its ED closes, homeless patients are disproportionately affected. Local hospitals were found to experience significantly increased volumes of homeless patients when compared to housed patients, with the marginally closer hospital more affected by this change.

## Figures and Tables

**Figure:**
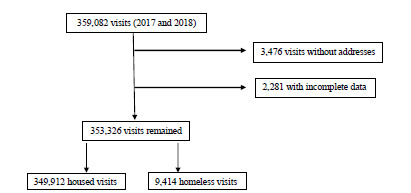
Flow chart of eligible total visits.

**Table 1:** Demographic information for housed vs homeless participants at The Miriam Hospital and Rhode Island Hospital.

Demographics	Housed (N = 343,912)	Homeless (N = 9,414)
Age (years)	50.03 (21.08)	45.14 (12.49)
Male	47.00%	73.15%
Race		
White	63.61%	54.90%
Black	13.98%	27.35%
Other	22.41%	17.75%
Hispanic/Latino	21.69%	16.19%
Health insurance		
Managed Medicaid	46.44%	67.62%
RI Medicaid	1.93%	6.86%
Medicare	16.97%	13.63%
Private insurance	19.68%	1.02%
Other	14.98%	10.87%

*RI*, Rhode Island.

**Table 2:** Emergency department visit characteristics for housed vs homeless patients at Rhode Island Hospital and The Miriam Hospital.

	Housed (N = 343,912)	Homeless (N = 9,414)
Arrival by EMS	30.42%	52.61%
Frequency of disposition		
Admission	27.70%	13.77%
Discharge	64.68%	74.03%
Average minutes to disposition		
Admission	304.0	466.0
Discharge	263.7	397.9
Chief complaint		
Abdominal pain	11.16%	4.73%
Chest pain	8.95%	5.39%
Back pain	4.69%	3.25%
Alcohol-related	2.94%	29.66%
Suicidal	1.43%	8.61%

*EMS*, emergency medical services.

**Table 3:** Total unique patients seen in Rhode Island Hospital and The Miriam Hospital emergency departments in 2017 and 2018, showing percent change after closure of Memorial Hospital.

	2017	2018	% Change
Combined			
Total unique patients	113,925	117,167	2.85%
Unique homeless patients	1,345	1,553	15.46%
Unique housed patients	112,580	115,614	2.69%
Homeless as % of total	1.18%	1.32%	11.86%
Rhode Island Hospital			
Total unique patients	66,591	65,563	−1.54%
Unique homeless patients	1,066	1,152	8.07%
Unique housed patients	65,525	64,411	−1.70
Homeless as % of total	1.60%	1.76%	10.00%
The Miriam Hospital			
Total unique patients	47,334	51,604	9.02%
Unique homeless patients	279	401	43.72%
Unique housed patients	47,055	51,203	8.82%
Homeless as % of total	0.59%	0.78%	32.30%
